# A meta-analysis of traditional and functional end-to-side anastomosis in radiocephalic fistula for dialysis access

**DOI:** 10.1007/s11255-020-02691-9

**Published:** 2021-01-02

**Authors:** Tang Weigang, Xu Wei, Gong Lifeng, Lu Jingkui, Li Yani, Jiang Huaqin, Li Hui

**Affiliations:** 1 Department of Nephrology, Wujin Hospital Affiliated with Jiangsu University, Changzhou City, Jiangsu Province China; 2 Department of Nephrology, The Wujin Clinical College of Xuzhou Medical University, Changzhou City, Jiangsu Province China

**Keywords:** Arteriovenous fistula, Functional end-to-side anastomosis, Dialysis access, Meta-analysis

## Abstract

**Objective:**

Functional vein end to arterial side (ETS) anastomosis uses vein side to arterial side (STS) anastomosis with distal vein ligation, which can achieve similar effects as those of ETS after STS anastomosis. The purpose of the study was to provide a meta-analysis to compare the clinical outcomes between traditional and functional ETS anastomosis in radiocephalic fistula for dialysis access.

**Methods:**

Databases including PubMed, EMbase, the Cochrane Library, CNKI, Wanfang database were searched from the inception to February 6, 2020. Eligible studies comparing traditional and functional ETS anastomosis in radiocephalic fistula were included. Data were analyzed using Review Manager Version 5.3.

**Results:**

Seven studies were included in the meta-analysis. Five randomized controlled trials and two cohort studies involving 841 patients were identified. Compared with traditional ETS anastomosis, functional ETS anastomosis had shorter anastomosis time (MD − 9.54, 95% CI − 17.96 to − 1.12, *P* = 0.03), higher surgical success rate (OR 3.80, 95% CI 1.76–8.22, *P* < 0.01), fewer complications(OR 0.18, 95% CI 0.08–0.39, *P* < 0.01), higher patency rate after 3 months (OR 4.91, 95% CI 1.19–20.33, *P* = 0.03), higher patency rate after 6 months (OR 1.90, 95%CI 1.09–3.31, *P* = 0.02), higher patency rate after 12 months (OR 1.70, 95% CI 1.09–2.66, *P* = 0.02). There was no difference after the two arteriovenous (AVF) anastomosisl methods concerning AVF maturation time (SMD − 0.48, 95% CI − 1.30–0.34, *P* = 0.25) and patency rate after 1 month (OR 1.77, 95% CI 0.65–4.80, *P* = 0.26).

**Conclusion:**

Functional ETS anastomosis had advantages of easy operation, high surgical success rate, few complications, high patency rate of 3 months and long-term, but did not have obvious advantage in the early stages concerning AVF maturation time and 1-month patency rate.

## Introduction

Autogenous arteriovenous fistula (AVF) is the preferred method of choice to achieve vascular access for long-term hemodialysis, because it has a low rate of complications and increases overall survival compared to other types of vascular access [1–7]. The radiocephalic fistula is considered first in the establishment of arteriovenous fistula [8]. The common types of AVF anastomosis used in uremic patients are vein end to arterial end (ETE), vein side to arterial end (STE), vein end to arterial side (ETS), and vein side to arterial side (STS) [9]. In clinical practice, ETS anastomosis is a most common type because of higher proximal venous flow, longer fistula survival and lesser long-term complications [10, 11]. European Society for Vascular Surgery (ESVS) guidelines also recommend ETS anastomosis [12].

In recent years, some scholars reported a modified AVF anastomosis, which had a good result. This modified AVF anastomosis was named functional ETS anastomosis using side-to-side anastomosis with distal vein ligation, which achieved similar effects as those of ETS after STS anastomosis. Our meta-analysis was conducted to compare the clinical outcomes between traditional and functional ETS anastomosis in radiocephalic fistula for dialysis access.

## Materials and methods

### Search strategy

Our meta-analysis has been reported in line with PRISMA (Preferred Reporting Items for Systematic Reviews and Meta-Analyses) and AMSTAR (Assessing the methodological quality of systematic reviews) Guidelines. Our meta-analysis was registered at the International Prospective Register of Systematic Reviews (Registration number: CRD42020166331).

We searched PubMed, Embase, the Cochrane Library, CNKI (China National Knowledge Infrastructure) and Wanfang from the inception to February 6, 2020. The combined text and MeSH terms included Arteriovenous Fistula, AVF, Access, Hemodialysis, End to Side, Functional, Modified. In addition, the cited papers and relevant references were searched manually to identify eligible studies. There were no language restrictions.

### Inclusion and exclusion criteria

**Fig. 1:**
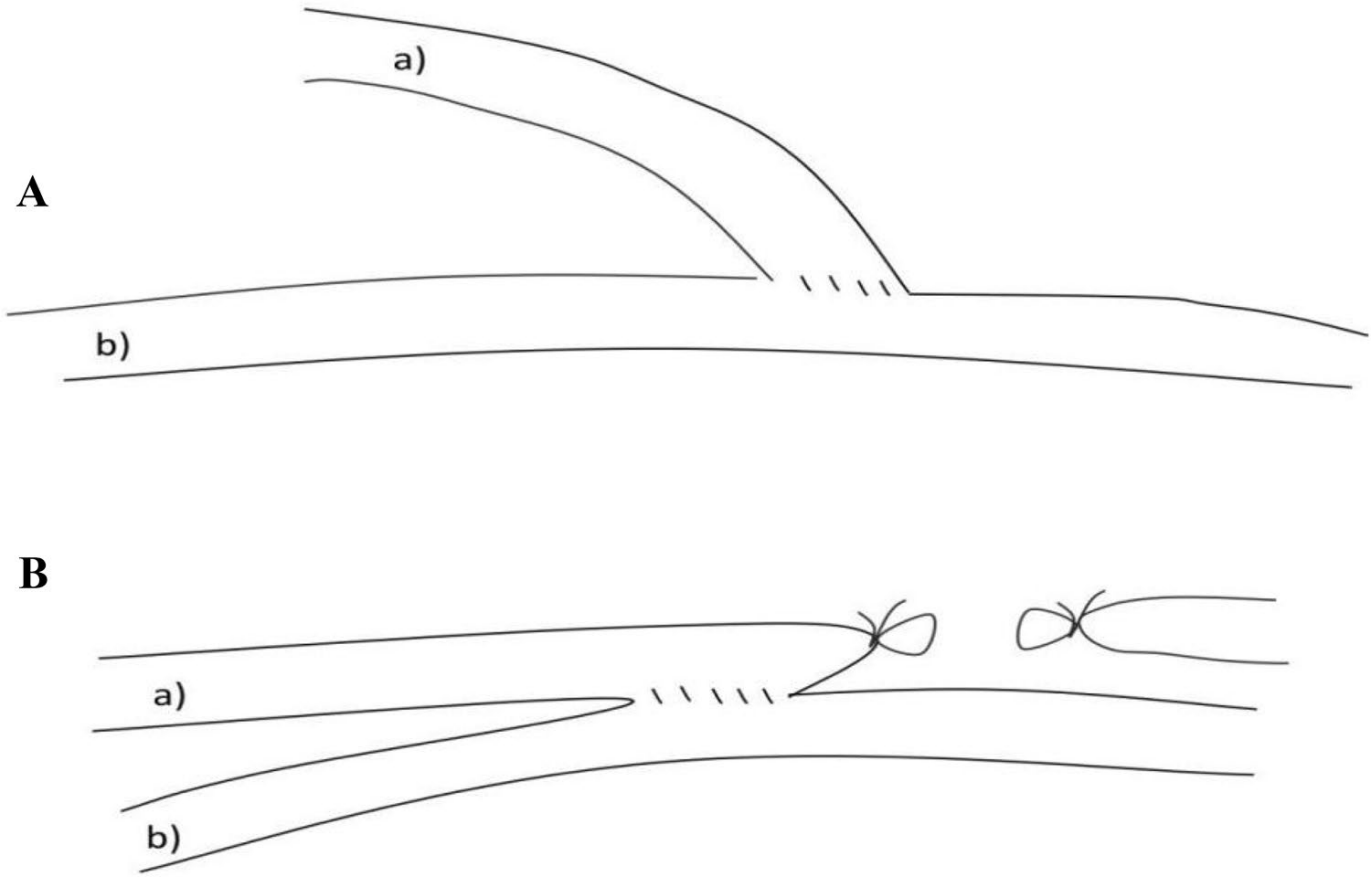
**a** Traditional ETS anastomosis uses vein end to arterial side anastomosis. **b** Functional ETS anastomosis uses side-to-side anastomosis with distal vein ligation. a cephalic vein; b radial artery

The inclusion criteria: (1) Randomized controlled trials (RCTs), cohort or case–control studies; (2) Hemodialysis patients; (3) Studies that compared traditional and functional ETS anastomosis in radiocephalic fistulas; Functional ETS anastomosis using side-to-side anastomosis with distal vein ligation (Fig. 1); (4) The main endpoint of the review was AFV patency rates. Secondary endpoints were anastomosis time, successful rate of surgery, maturation time, complications.

The exclusion criteria: (1) Severe cardiac insufficiency or poor conditions of vascular; (2) Case series, comments, reviews; (3) Lack of relevant outcomes data.

### Data extraction and quality assessment

Data were extracted independently by two investigators using standard data extraction forms. In the case of disagreement, a third investigator was consulted. We extracted characteristics including first author, year of publication, location, study design, follow-up period, age, sex, sample size, anastomosis time, successful rate of surgery (The primary fistula patency was defined as the immediate success rate of surgery), maturation time of AVF (AVF maturation was defined as venous diameter > 0.6 cm and access flow > 600 mL/min [1]), complications (Hemorrhage, infection, hand swelling, thrombosis, venous stenosis), patency rate (The primary patency rate was defined as the unassisted patency rate without endovascular and open surgery; The secondary patency rate was defined as the assisted patency rate with salvage procedures). The Cochrane assessment tool was used to evaluate the quality of RCTs [13]. The Newcastle–Ottawa scale (NOS) was used to evaluate the quality of non-randomized studies [14].

### Statistical analysis

We performed the data analysis using Review Manager Version 5.3 (Cochrane Collaboration). Heterogeneity between studies was assessed by using *I*^2^ statistics. We considered *I*^2^ > 50% and *P* < 0.10 to imply significant heterogeneity [15]. Homogeneous data was performed using the fixed-effects model. Heterogeneous data was performed using the random-effects model. We presented categorical variables as Odds Ratios (OR). Continuous data were presented as the mean difference (MD). Summary estimates and 95% confidence intervals (CIs) were calculated. Overall effects were determined by the using *Z*-test. A *P* value < 0.05 was considered significant. Publication bias was assessed using sensitivity analysis.

## Results

### Study selection and characteristics

**Fig. 2:**
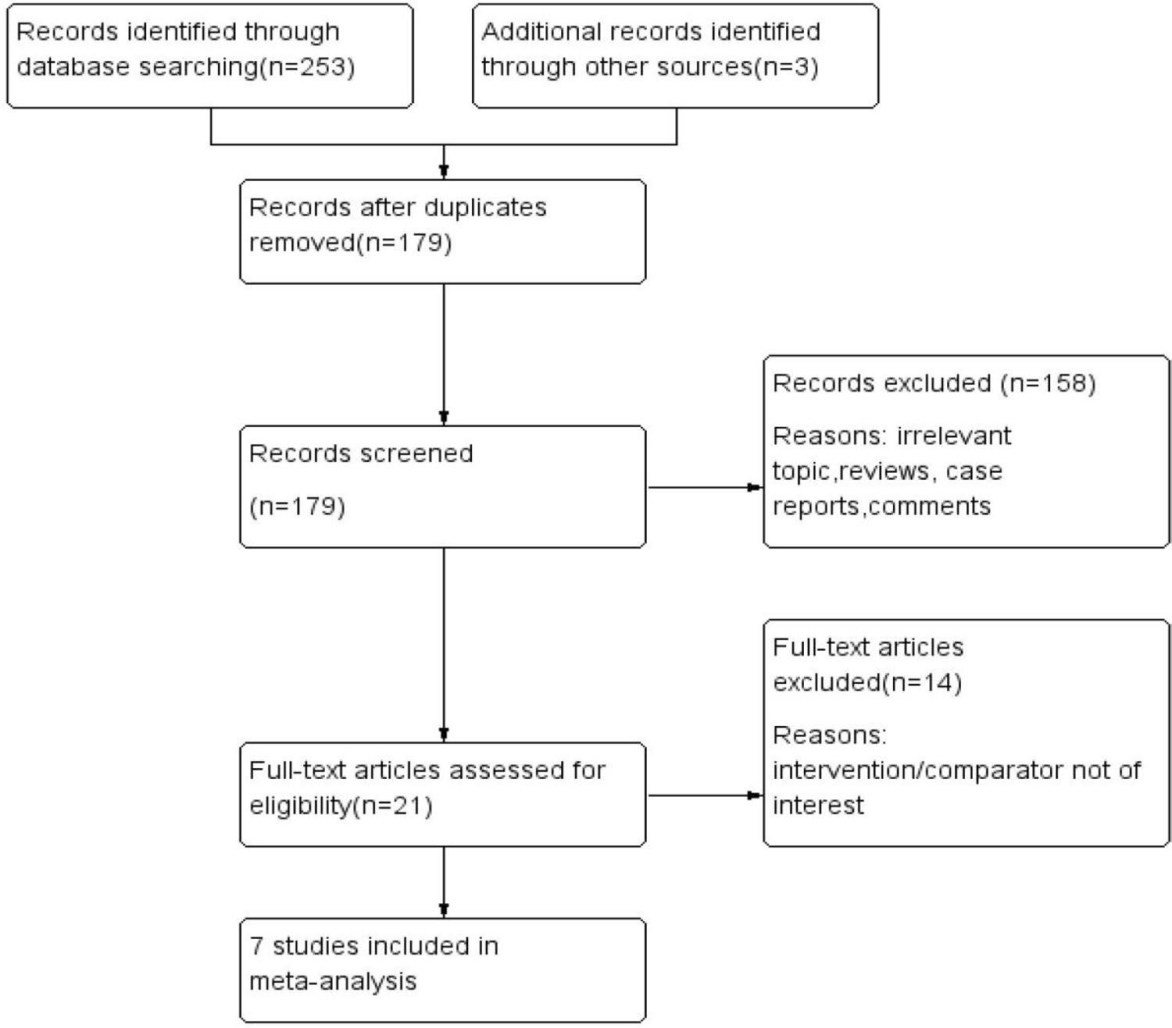
Flow diagram of the literature search

A flow diagram of the selection process is shown in Fig. 2. Finally, six studies from China [16–21] and one study from America [22] were included in this analysis. Of the seven studies, five were RCTs and two were cohort studies. Five studies were published in the Chinese journal. As a whole, 458 patients were included in the functional ETS anastomosis group and 383 patients were included in the traditional ETS anastomosis group. The follow-up period was from 6 months to 2 years. The risk of bias in included RCTs was moderate. The cohort studies achieved scores of ≥ 6 points, which were considered to be of high quality. The baseline characteristics of these studies are listed in Tables 1. The NOS assessment is listed in Table 2 and the Cochrane assessment is listed in Table 3.

**Table 1:** Characteristics of the included studies

Study (year)	Sample size	Mean age (years)	Male/Female	Diabetic nephropathy (%)	Scr (μmol/L)	Bun (mmol/L)	HB (g/L)	Surgical site	Vein size	The size of the anastomosis
Guan Sheng (2010)	Experimental group: 63 Traditional group:61	60.2 ± 12.5 62.1 ± 15.2	36/27 32/29	–	1008.6 ± 456.2 998.6 ± 500.2	36.5 ± 12.9 39.5 ± 15.1	89.2 ± 12.5 86.1 ± 15.8	Radiocephalic fistulas on the snuff nest or wrist	≥ 1.6 mm	5–8 mm 5–8 mm
Zhang Caixia (2017)	Experimental group: 70 Traditional group:70	61.5 ± 11.1 62.2 ± 9.8	40/30 42/38	25(35.7%) 20(28.6%)	689.5 ± 154.9 702.4 ± 163.4	–	92.7 ± 19.0 89.2 ± 21.1	Radiocephalic fistulas on the wrist	–	5–8 mm 5–8 mm
Tang Weigang (2019)	Experimental group:110 Traditional group:40	56.8 ± 14.8 57.5 ± 15.1	61/49 23/17	–	–	29.3 ± 11.7 30.2 ± 12.4	89.1 ± 13.6 87.4 ± 15.8	Radiocephalic fistulas on the forearm	24 patients < 2.5 mm 8 patients < 2.5 mm	6–10 mm 5–6 mm
Xu Hui (2017)	Experimental group:60 Traditional group:60	59.7 ± 9.2 60.5 ± 10.3	34/26 35/25	–	999.5 ± 477.4 1002.3 ± 491.2	37.9 ± 13.2 38.5 ± 14.5	88.5 ± 12.7 87.6 ± 13.5	Radiocephalic fistulas on the wrist	≥ 1.6 mm	5–8 mm 5–8 mm
Chen Junzhu (2018)	Experimental group:40 Traditional group:40	58.3 ± 8.5 58.3 ± 8.5	27/13 24/16	13(32.5%) 14(41.4%)	948.29 ± 361.65 923.43 ± 372.43	36.8 ± 14.8 36.2 ± 15.2	85.7 ± 12.1 86.4 ± 11.4	Radiocephalic fistulas on the snuff nest or wrist	≥ 1.6 mm	6–7 mm 6 mm
Sun Yibing (2014)	Experimental group:83 Traditional group:83	55.3 ± 6.4	96/70	–	–	–	–	Radiocephalic fistulas on the wrist	–	7–8 mm –
O'Banion (2015)	Experimental group:32 Traditional group:29	58 ± 12.5 55 ± 14.6	25/7 19/10	22(68%) 22(76%)	–	–	–	Radiocephalic fistulas on the forearm	1.9 ± 0.59 mm 2.6 ± 0.68 mm	1.3–1.5 cm –

**Table 2:** Quality assessment of cohort studies

Studies	Selection	Comparability	Outcome	Score
Tang Weigang 2019	★★★	★	★★★	7
O’Banion 2015	★★★	★	★	6

**Table 3:** Risk of bias of randomized control trial

Study	Random sequence generation	Allocation concealment	Blinding of participants and personnel	Incomplete outcome data	Selective reporting	Other bias
Zhang Caixia (2017)	Low risk	Unclear	Unclear	Low risk	Low risk	Unclear
Xu Hui (2017)	Low risk	Low risk	Low risk	Low risk	Low risk	Unclear
Guan Sheng (2010)	Low risk	Low risk	Low risk	Low risk	Low risk	Unclear
Chen Junzhu (2018)	Low risk	Unclear	Unclear	Low risk	Low risk	Low risk
Sun Yibing (2014	Unclear	Unclear	Unclear	Low risk	Low risk	Unclear

### Meta-analysis results

**Fig. 3:**
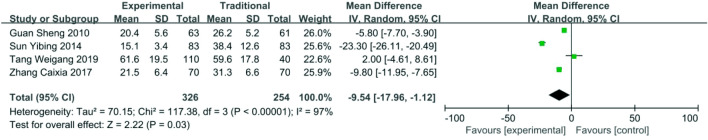
Forest plot of anastomosis time between functional ETS group and traditional ETS group. Control group: traditional ETS anastomosis; Experimental group: functional ETS anastomosis

*Anastomosis time *Data about anastomosis time were reported in four articles [16–18, 21]. There was significant heterogeneity among the studies (*P* <0.01, *I*^2^ = 97%), so finally the random-effects model was used for the meta-analysis. Anastomosis time of the functional ETS group was shorter than the traditional STE group (MD − 9.54, 95% CI − 17.96 to − 1.12, *P* = 0.03) (Fig. 3).

**Fig. 4:**
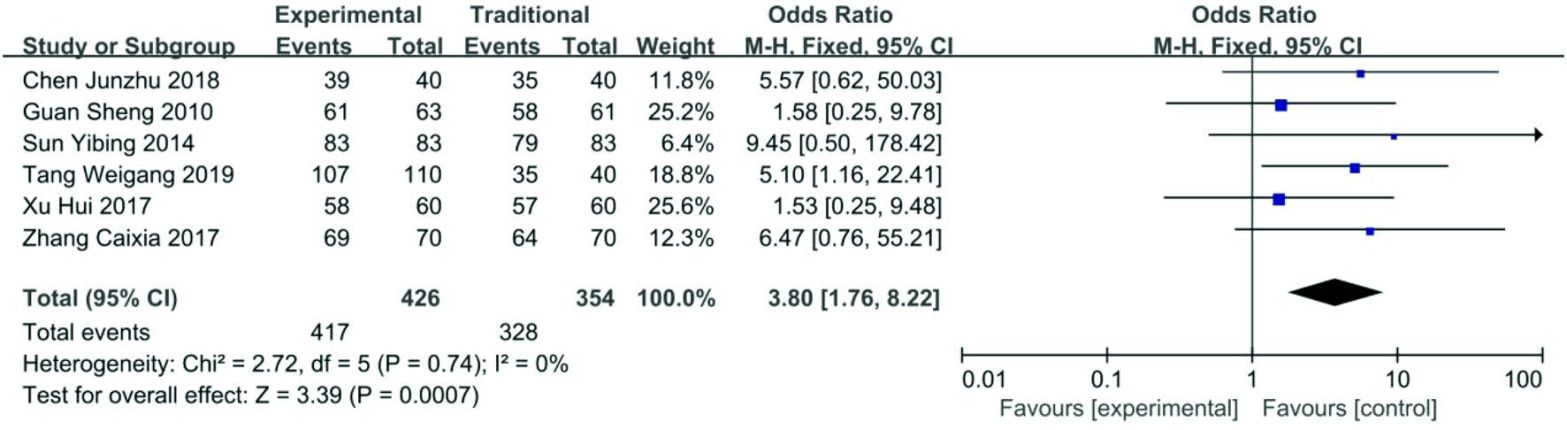
Forest plot of succesful rate of surgery between functional ETS group and traditional ETS group. Control group: traditional ETS anastomosis; Experimental group: functional ETS anastomosis

*Succesful rate of surgery* Data about successful rate of surgery were reported in six articles [16–21], 417/426 (97.9%) for the functional ETS group and 328/417 (92.7%) for the traditional ETS group. The heterogeneity among these studies was not substantial (*P* = 0.74, *I*^2^ = 0%), so the fixed-effects model was used for the meta-analysis. Succesful rate of surgery in the functional STE group was higher than the traditional ETS group (OR 3.80, 95% CI 1.76–8.22, *P* < 0.01) (Fig. 4).

**Fig. 5:**
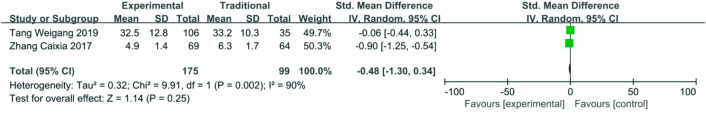
Forest plot of maturation time of AVF between functional ETS group and traditional ETS group. Control group: traditional ETS anastomosis; Experimental group: functional ETS anastomosis

*Maturation time of AVF* Data about maturation time of AVF were reported in two articles [17, 18]. There was significant heterogeneity between the two studies (*P* < 0.01, *I*^2^ = 90%), so finally the random-effects model was used for the meta-analysis. There was no significant difference between both groups concerning maturation time of AVF (SMD − 0.48, 95%CI − 1.30−0.34, *P* = 0.25) (Fig. 5).

**Fig. 6:**
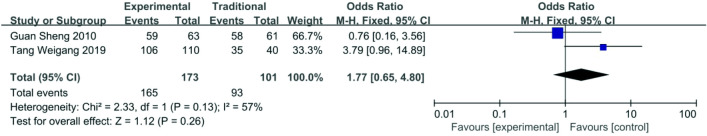
Forest plot of patency rate after 1 month between functional ETS group and traditional ETS group. Control group: traditional ETS anastomosis; Experimental group: functional ETS anastomosis

*Patency rate after 1 month *Data about patency rate after 1 month were reported in two articles [16, 18], 165/173 (95.4%) for the functional ETS group and 93/101 (92.0%) for the traditional STE group. The heterogeneity between the two studies was not substantial (*P* = 0.13, *I*^2^ = 57%), so the fixed-effects model was used for the meta-analysis. There was no significant difference between both groups concerning patency rate after 1 month (OR 1.77, 95% CI 0.65–4.80, *P* = 0.26) (Fig. 6).

**Fig. 7:**
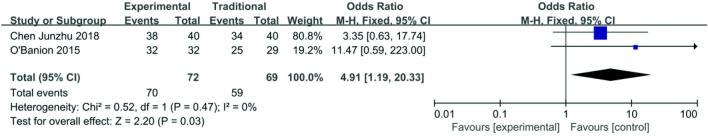
Forest plot of patency rate after 3 month between functional ETS group and traditional ETS group. Control group: traditional ETS anastomosis; Experimental group: functional ETS anastomosis

*Patency rate after 3 months *Data about patency rate after 3 months were reported in two articles [20, 22], 70/72 (97.2%) for the functional ETS group and 59/69 (85.5%) for the traditional ETS group. The heterogeneity between the two studies was not substantial (*P* = 0.47, *I*^2^ = 0%), so the fixed-effects model was used for the meta-analysis. Patency rate after 3 months in the functional ETS group was higher than the traditional ETS group (OR 4.91, 95% CI 1.19–20.33, *P* = 0.03) (Fig. 7).

**Fig. 8:**
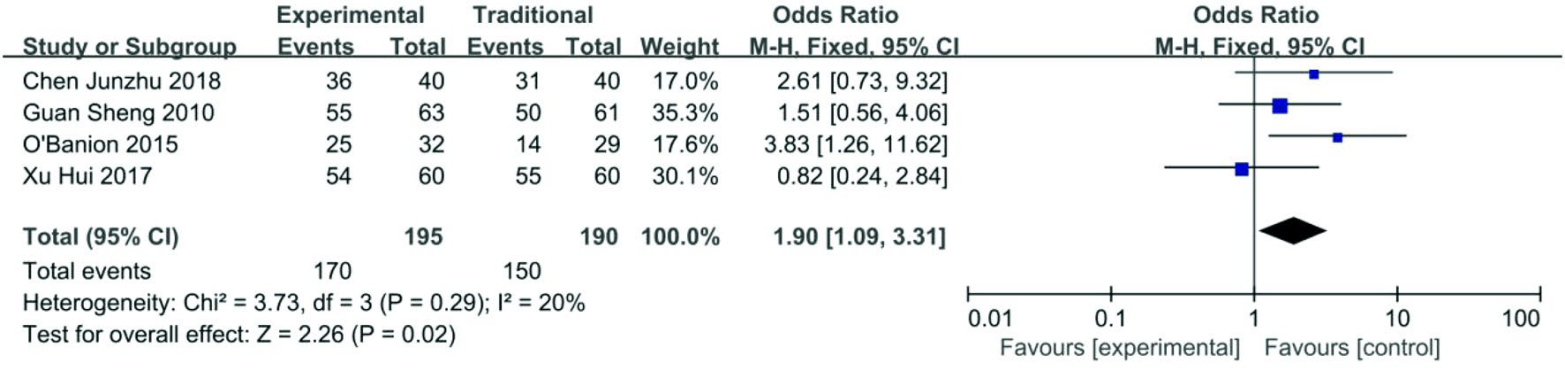
Forest plot of patency rate after 6 month between functional ETS group and traditional ETS group. Control group: traditional ETS anastomosis; Experimental group: functional ETS anastomosis

*Patency rate after 6 months *Data about patency rate after 6 months were reported in four articles [16, 19, 20, 22], 170/195 (87.1%) for the functional STE group and 150/190 (78.9%) for the traditional ETS group.The heterogeneity among these studies was not substantial (*P* = 0.29, *I*^2^ = 20%), so the fixed-effects model was used for the meta-analysis. Patency rate after 6 months in the functional ETS group was higher than the traditional ETS group (OR 1.90, 95% CI 1.09–3.31, *P* = 0.02) (Fig. 8).

**Fig. 9:**
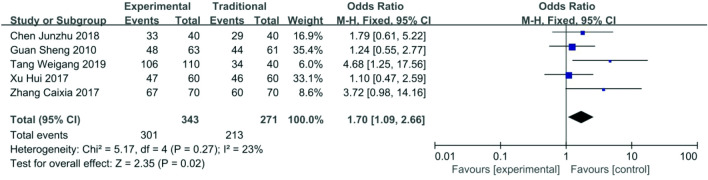
Forest plot of patency rate after 12 months between functional ETS group and traditional ETS group. Control group: traditional ETS anastomosis; Experimental group: functional ETS anastomosis

*Patency rate after 12 months *Data about patency rate after 12 months were reported in five articles [16–20], 301/343 (87.7%) for the functional ETS group and 213/271 (78.6%) for the traditional ETS group. The heterogeneity among these studies was not substantial (*P* = 0.27, *I*^2^ = 23%), so the fixed-effects model was used for the meta-analysis. Patency rate after 12 months in the functional ETS group was higher than the traditional ETS group (OR 1.70, 95% CI 1.09–2.66, *P* = 0.02) (Fig. 9).

**Fig. 10:**
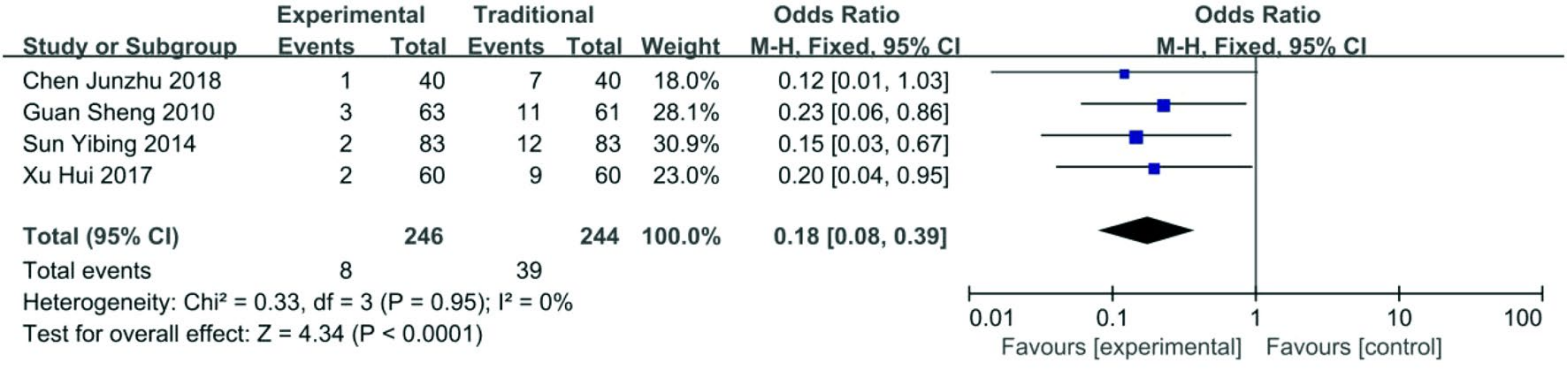
Forest plot of complications between functional ETS group and traditional ETS group. Control group: traditional ETS anastomosis; Experimental group: functional ETS anastomosis

*Complications *Data about complications were reported in four articles [16, 19–21]. Incidences of hemorrhage (1.40%, 2/143), infection (1.40%, 2/143), hand swelling (0.55%, 1/183), early thrombosis (1.00%, 1/101),thrombosis (2.11%, 3/142), venous stenosis (0%, 0/110) in the functional ETS group were all lower than incidences of hemorrhage (6.99%, 10/143), infection (6.29%, 9/143), hand swelling (1.09%, 2/183),early thrombosis (10.10%, 10/99), thrombosis (11.27%, 16/142), venous stenosis (3.96%, 4/101) in the traditional ETS group. The total incidence of complications were 8/246 (3.3%) for the functional ETS group and 39/244 (15.9%) for the traditional ETS group. The heterogeneity among these studies was not substantial (*P* = 0.95, *I*^2^ = 0%), so the fixed-effects model was used for the meta-analysis. Complications of the functional ETS group were fewer than the traditional ETS group (OR 0.18, 95% CI 0.08–0.39, *P* < 0.01) (Fig. 10).

### Publication bias

For heterogeneous studies, the number of included studies (less than 3) was too small to conduct any sufficient analysis of publication bias. Remarkably, The majority of participants in O’Banion study were not Asian, which was different from other studies. If we deleted O’Banion study, patency rates after 3 months (*P* = 0.15) and 6 months (*P* = 0.23) were no difference between the two groups.

## Discussion

The well-functioning AVF provides enough blood flow without complications for a long time, which is the key to successful hemodialysis. Considering the limitation of vascular resources, the radiocephalic fistula is the most common choice of initial vascular access. The most commonly reported techniques of AVF anastomosis are ETE, STE, ETS and STS.ETE and STE anastomosis has the low fistula flow rate [23], which are used less in clinical practice.STE anastomosis also has the greatest risk of venous hypertension [24]. ETS anastomosis is a highly recommended anastomosis type because it has a high fistula flow and the low risk of venous hypertension of the hand [25]. STS anastomosis is the easiest construct technique and used commonly, which has the highest fistula flow [24]. However, STS anastomosis has the highest risk of venous hypertension. The most effective solution of venous hypertension is ligation of the distal venous arm [24]. The procedure of functional ETS anastomosis is STS anastomosis followed by distal cephalic vein ligation or distal cephalic vein ligation followed by STS anastomosis. It has a similar effect of traditional ETS anastomosis and has advantages of STS anastomosis at the same time. Sung and Zafar-Ul reported that one-year patency rates of AVF using functional STE anastomosis were both nearly 90% [26, 27]. Sun Yibing reported that two years patency rate of AVF using functional ETS anastomosis achieved 90% [21]. Our meta-analysis revealed that there was no difference between the two AVF anastomosis methods concerning maturation time and 1-month patency rate. Compared with the traditional ETS anastomosis, the advantages of the functional ETS anastomosis were shorter anastomosis time, higher surgical success rate, 3–12 months patency rate.

The recognized factors affecting the maturation and patency of AVF are reported to venous blood flow and the size of the vein [28, 29]. Sufficient venous blood flow and vein sizes will lead to reduce the risk of thrombosis, promote early maturation and increase the patency rate. If the fistula does not develop early thrombosis within three months, the risk of overall thrombosis is decreasing drastically [30]. Creating an fistula with early maturation and patency can reduce usage time of hemodialysis catheter, hospital stays and overall cost. In the procedure of functional ETS anastomosis, using STS anastomosis can reduce the impact of vein size and create large anastomotic diameter, which allows for larger venous blood flow. In the studies of Tang Weigang and O'Banion, the vein size in functional ETS anastomosis group were obviously smaller than in traditional ETS anastomosis group, but anastomotic diameter in functional ETS anastomosis group were obviously larger than in traditional ETS anastomosis group [18, 22]. In the traditional ETS anastomosis, some veins of small inner diameter cannot achieve ideal anastomotic caliber even after mechanical expansion and bevel pruning. The traditional ETS anastomosis is limited by the diameter of the vein. This explains that functional ETS anastomosis had higher 3 month patency rate and simultaneously long-term (6–12 months) patency rate. In our meta-analysis, average maturation time in functional ETS anastomosis was shorter than in traditional ETS anastomosis and 1-month patency rate in functional ETS anastomosis was higher than in traditional ETS anastomosis. However, there was no statistical difference concerning maturation time and 1-month patency. For the analysis about maturation time and 1-month patency rate, the number of included studies were only two, which might not be adequate to judge conclusion.

Many operators of functional ETS anastomosis summarize the experience of operation in their studies. First, the operator of functional ETS anastomosis need not turnover vessel repeatedly because the position of arteries and veins is relatively stationary and paralleled, which can reduce arteriovenous angulation and reduce risk of poor arteriovenous alignment, vascular distortion and rotation. Second, the operator of functional ETS anastomosis need not pruner anastomotic surface of vein overmuch, which can save part of the operation time and reduce the damage to the vessel [17–21]. These two points can also explain that the functional ETS anastomosis reduces the risk of thrombosis, has higher surgical success rate and improve the patency rate. Furthermore, in the case of postoperative AVF thrombosis, we can open the ligation line of the distal vein to explore and remove thrombosis.

There were some limitations in our meta-analysis. First, patency rates in most of the included studies were not defined clearly as primary or secondary patency rate. One year AVF patency rate of functional ETS anastomosis in our meta-analysis were 87.7%. The Tang’s study performed in our hospital was a clue that one-year primary patency rate of the functional ETS anastomosis was 96.4% (106/110) [18]. Second, gender, age, diabetes mellitus are also reported to be the risk factors affecting the function of AVF. These risk factors were not different between the two anastomosis methods in most studies. However, these risk factors were not counted in the few studies we do not know if there’s a difference. Third, for the analysis about maturation time and 1–3 months patency rate, included studies had a small sample size, so our meta-analysis may not be adequate to judge the accuracy. Fourth, in the O’Banion study, the majority of participants were not Asian and patency rate after 6 months in the traditional ETS anastomosis group was only 48%. If we deleted O’Banion study, 1–6 month patency rates were all no difference between the two groups. So publication bias was still possible.

## Conclusions

Functional ETS anastomosis has a similar effect of traditional ETS anastomosis and has advantages of STS anastomosis at the same time. Our meta-analysis revealed that the advantages of functional ETS anastomosis were an easy operation, high surgical success rate, few complications, high patency rate of 3 months and long-term. AVF maturation time and 1-month patency rate of the two anastomosis methods might be similar. To further confirm the conclusion, more large multicenter randomized controlled trials comparing the two AVF anastomosis methods are necessary.
